# What explains gender inequality in HIV infection among high-risk people? A Blinder-Oaxaca decomposition

**DOI:** 10.1186/s13690-021-00758-2

**Published:** 2022-01-04

**Authors:** Mansour Sajadipour, Satar Rezaei, Seyed Fahim Irandoost, Mohammadreza Ghaumzadeh, Mohamadreza Salmani nadushan, Mohammad Gholami, Yahya Salimi, Zahra Jorjoran Shushtari

**Affiliations:** 1 Department of Health, South Tehran health center, Tehran University of Medical Sciences, Tehran, Iran; 2 Social Determinants of Health Research Center, Clinical Research Institute, Urmia University of Medical Sciences, Urmia, Iran; 3 Department of Medical Microbiology, Aja University of Medical Sciences, Tehran, Iran; 4 Social Development and Health Promotion Research Center, Health Institute, Kermanshah University of Medical Sciences, Kermanshah, Iran; 5 Social Determinants of Health Research Center, University of Social Welfare and Rehabilitation Sciences, Tehran, Iran

**Keywords:** Gender inequality, HIV infection, High-risk people, Blinder-Oaxaca decomposition

## Abstract

**Background:**

Despite clear evidence on role of gender in vulnerability and exposure to HIV infection, information on gender-related inequalities in HIV and related factors are rarely documented. The aim of this study was to measure gender inequality in HIV infection and its determinates in Tehran city, the capital of Iran.

**Methods:**

The study used the data of 20,156 medical records of high-risk people who were admitted to Imam Khomeini Voluntary Counseling and Testing site in Tehran from 2004 to 2018. The Blinder-Oaxaca decomposition was used to quantify the contribution of explanatory variables to the gap in the prevalence of HIV infection between female and male.

**Results:**

The age-adjusted proportion of HIV infection was 9.45% (95%Cl: 9.02, 9.87). The absolute gap in the prevalence of HIV infection between male and female was 4.50% (95% CI: − 5.33, − 3.70%). The Blinder-Oaxaca decomposition indicated that most explanatory factors affecting the differences in HIV infection were job exposure, drug abuse, history of imprisonment, injection drug, heterosexual unsafe sex, and having an HIV-positive spouse.

**Conclusion:**

The results can provide evidence for health policymakers to better planning and conducting gender-based preventive and screening programs. Policies aiming at promoting HIV preventive behaviors among male may reduce the gap in HIV infection between female and male in Iran.

## Background

The human immunodeficiency virus (HIV) continues as one of the serious public health challenges in developing countries. According to the Global Burden of Disease Study, the annual incidence of HIV infection was about 2.6 million per year in 2015 [1]. About 37.7 million people living with HIV/AIDS in 2020 globally [2]. Of these, about 6.1 million infected people with HIV are not aware of their infection [3]. In 2020, 5.8 million people were living with HIV in Asia and the Pacific regional [3]. Estimation shows that the prevalence of HIV among the Iranian population age ≥ 18 was 90 per 100,000 people (15,000 female and 39,000 male) in 2019 [4]. The incidence of HIV infected cases in all age groups in Iran was estimated 2.86 per 100,000 people [4]. Although HIV can affect people regardless of sexual orientation, race, ethnicity, gender, or age, some people are at higher risk for HIV than others and need special consideration because of their socio-demographic characteristics and risk factors.

Gender is one of the important social determinants of health which has an important role in health equity and well-being [5, 6]. “Gender is defined as a social concept that differentiates the power, roles, responsibilities, and obligations of women from that of men in society” [7]. Gender inequalities in health are manifested in a context in which access to, utilization of healthcare services, morbidity, and mortality differed preventable and unnecessarily between women and men in a society [8]. Gender inequalities affect health outcomes through various pathways. Some health outcomes are determined primarily by biological sex differences [9]. Other health outcomes are the results of socialization of the gender roles and gendered power relations supported by social norms about masculinity and femininity in societies. Gender usually is a stable social determinant of health during life-course, whereas some other social determinants such as education, income, and occupation could change during people’s life. Female gender through economic dependency, intimate partner violence, and lack of power to negotiate safe sex can increase the risk of HIV infection [10–13]. Evidence shows that women are disproportionally affected by HIV around the world [7, 14]. This subpopulation may be more at risk, less detected, and less likely to receive timely and appropriate health care services and treatments. This situation makes reaching the 90–90-90 target, to control HIV infection hard. In addition, according to the literature, not only there is a gender inequality in health outcomes such as HIV, but also social consequences related to such health problems such as social stigma, coping, and social support differs among people based on their gender [14, 15]. As HIV related stigma has a strong negative effect on female, and men differently cope with stressful condition such as HIV status than female [16]. Despite the evidence highlighted on the role of gender in HIV infection, there is little empirical evidence about the role of gender difference as the main source of HIV infection inequality and its responsible factors among the developing countries [7, 10, 14, 17]. It seems that HIV infection is disproportionally distributed by gender in Tehran, therefore we seek to examine this gender disparities and its determinants among people who refer to a behavioral consultation center in Tehran. Knowing about the magnitude of gender inequality and its determinants may provide an insight about gender distribution of the HIV infection that is necessary for effective HIV prevention programing and managing the limited resources for health planners.

## Methods

The data used in this study come from 20,156 medical records of high-risk people who were admitted to Imam Khomeini Voluntary Counseling and Testing (VCT) site of Tehran University of Medical Sciences from 2004 to 2018. The Imam Khomeini VCT is among major site that provides free counseling, testing, and treatment services for people with high-risk behaviors, HIV-positive and patient with AIDS to cope with the test result and avoid high-risk behavior. The people who are identified as HIV positive will be given more information, post-testing counseling, and treatment. The routine data in the VCT were collected using a checklist that including some demographic data, behavioral information, and personal history.

HIV testing was performed at the VCT laboratory using a standard western blot test was performed to confirm HIV status. The outcome variable in this study was the HIV test result (0, no; 1, yes).

This study was approved by the Ethics Committee of Kermanshah University of Medical Sciences (IR.KUMS.REC.1398.364).

### Statistical analysis

There were some missing data in the variables, therefore, we used Multiple Imputation (MI) to account for the missing values and assumed that the data were not missing completely at random.

The imputed data sets were analyzed using the MI suite of commands. The analyses were performed on 50 multiple imputed datasets. We used Blinder-Oaxaca (BO) decomposition with a logistic model [18, 19] to decompose the absolute difference in the HIV infection between female and male. In the BO model, the prevalence gap between female and male in HIV infection can be decomposed into two components: first, the percentage attributable to different levels of the explanatory variables between female and male (composition, endowment, or explained effect), and second the percentage attributable to explanatory variables having differential effects on HIV infection in female and male (response or coefficient effect). The following regression model linking the HIV infection, Y; to a set of predictors, x, eq. 1 and 2 are presented for female and male, respectively:

1 $$ YF=\beta Fxi+\varepsilon iF $$

2 $$ YM=\beta Mxi+\varepsilon iM $$

The difference between the mean values of HIV infection for the female, *yA*, and male, *yB*, can be calculated as:

3 $$ YM- YF=\Delta xBF+\Delta BxF+\Delta x\Delta \beta =E+C+ CE $$

where *xF* and *xM* are the average predictors for female and male, respectively; *βF* and *βM* denote the coefficients of predictors for female and male, respectively; and Δ*x* = *xF-xM* and Δ*β* = *βF*-*βM*. Based on eq. 3, the mean difference in the prevalence of HIV infection was divided into three components: first, the percentage attributable to different levels of predictors between female and male (explained components, *E*), second, the percentage attributable to predictors that have differential effects on HIV infection in female and male (the response or coefficient effect, *C*), and third, the percentage attributable to the interaction between the difference in the mean value of predictors and their coefficients (*CE*). The nonlinear BO decomposition method with a logistic model to decompose the gap in HIV infection between female and male [20]. The level of significance (alpha level) in all analyses was set at 0.05. All statistical analysis procedures were done using STATA 11(version 11; StataCorp, TX, USA) [21].

## Results

Table 1 present the descriptive characteristics of the study population included in the study. As indicated in Table 1, nearly 70% of the study participants were men; most participants were 20–40 years (65.22%), and rarely used the condom (56.85%). The majority of participants had academic education (54.35%). Only 21.86% were drug abuse, and 19.99% had a history of prison. Age-adjusted proportion of HIV infection was 9.45% (95%Cl: 9.02, 9.87). There was a higher proportion of age-adjusted proportion of HIV infection among the male (10.50%) compared to the female (7.15%).

**Table 1:** Characteristics of study participants who admitted to Imam Khomeini Voluntary Counseling and Testing by HIV infection, Tehran, Iran (2004–2018)

	HIV^**+**^	HIV^**−**^	All participants	Pvalue	Missing
	***N*** (%)	***N*** (%)	***N*** (%)		***N*** (%)
**Age groups**				< 0.001	99 (0.49)
< 20 y	62 (7.21)	798 (92.79)	860 (4.29)		
20–40 y	818 (6.26)	12,245 (93.74)	13,063 (65.22)		
40–60 y	1130 (20.91)	4274 (79.09)	5404 (26.98)		
> 60	129 (18.38)	573 (81.62)	702 (3.50)		
**Gender**				< 0.001	72 (0.36)
Male	1685 (12.01)	12,345 (87.99)	14,030 (69.95)		
Female	455 (7.55)	5571 (92.45)	6026 (30.05)		
**Marital status**				< 0.001	17,509 (86.99)
Single	250 (30.05)	582 (69.95)	1073 (40.97)		
Married	240 (22.37)	833 (77.63)	832 (31.77)		
Divorced	72 (15.96)	379 (84.04)	451 (17.22)		
Widowed	26 (55.32)	21 (44.68)	47 (1.79)		
Other	4 (1.85)	212 (98.15)	216 (8.25)		
**History of imprisonment**				< 0.001	8262 (41.05)
No	507 (5.34)	8987 (94.66)	9494 (80.01)		
Yes	1154 (48.65)	1218 (51.35)	2372 (19.99)		
**Drug abuse**				< 0.001	8411 (41.79)
No	427 (4.66)	8729 (95.34)	9156 (78.14)		
Yes	1208 (47.17)	1353 (52.83)	2561 (21.86)		
**Condom use**				< 0.001	12,174 (60.48)
Never	298 (13.48)	1913 (86.52)	2211 (27.80)		
Rarely	386 (8.54)	4136 (91.46)	4.522 (56.85)		
Ever	79 (6.47)	1142 (93.53)	1221 (15.35)		
**Job title**				< 0.001	281 (1.40)
Far from home	10 (8.47)	108 (91.53)	118 (0.59)		
Risky environment	20 (1.00)	1990 (99.00)	2010 (10.13)		
Official	967 (9.51)	9199 (90.49)	10,166 (51.22)		
Unemployed	1098 (18.15)	4953 (81.85)	6051 (30.49)		
Student	34 (2.26)	1468 (97.74)	1502 (7.57)		
**Educational level**				< 0.001	11,250 (55.89)
Illiterate	68 (40.72)	99 (59.28)	167 (1.88)		
Elementary	297 (45.00)	363 (55.00)	660 (7.43)		
Junior	450 (37.82)	740 (62.18)	1190 (13.40)		
High school	303 (14.88)	1733 (85.12)	2036 (22.93)		
Academic	154 (3.19)	4671 (96.81)	4825 (54.35)		
**Root of transmission**				< 0.001	72 (0.36)
Injection drug	1166 (35.18)	2148 (64.82)	3314 (16.52)		
Unsafe sex (heterosexual)	322 (3.36)	9272 (96.64)	9594 (47.84)		
Unsafe sex (homosexual)	37 (8.04)	423 (91.96)	460 (2.29)		
Blood and blood products	23 (37.70)	38 (62.30)	61 (0.30)		
Vertical transfer	50 (10.46)	428 (89.54)	478 (2.38)		
High risk spouse	77 (12.44)	542 (87.56)	619 (3.09)		
HIV ^+^ Spouse	205 (27.55)	539 (72.45)	744 (3.71)		
Job Exposure	5 (0.24)	2111 (99.76)	2116 (10.55)		
Unknown	255 (9.55)	2415 (90.45)	2670 (13.31)		

Table 2 shows the results from multiple logistic regression analysis for the association between HIV status and its determinants. There was a adjusted association between age and HIV infection (OR = 1.03, 95%CI: 1.01, 2.36). Male were more likely to get HIV infection than female (OR = 1.24, 95%CI: 1.05, 1.47). History of imprisonment and drug abuse significantly increased the odds of being HIV positive by nearly 80%. The unemployed people had a nearly four-fold increase in the odds of HIV infection compared to those who had a mobile job (OR = 3.74, 95% CI: 1.86, 7.50). People with an academic degree were less likely to have HIV infection compared to those who were illiterate (OR = 0.48, 95% CI: 0.34, 0.70).

**Table 2:** Multiple logistic regression results of determinants of HIV infection who admitted to Imam Khomeini Voluntary Counseling and Testing (*n* = 20,128), Tehran, Iran (2004–2018)

Variables	Crude OR	95% CI	Adjusted OR	95% CI
**Age**	1.05***	1.05, 1.06	1.03***	1.01, 1.05
**Gender**				
Female	ref	ref	ref	ref
Male	1.67***	1.50, 1.86	1.24**	1.05, 1.47
**Marital status**				
Married	ref	ref	ref	ref
Single	1.00	0.84, 1.20	0.89	0.75, 1.05
Widowed	1.53	0.96, 2.43	1.33	0.86, 2.05
Divorced	0.83***	0.68, 1.01	0.80**	0.65, 0.98
Other	0.62***	0.45, 0.87	0.59***	0.44, 0.79
**History of imprisonment**				
No	ref	ref	ref	ref
Yes	8.24***	7.41, 9.17	1.83***	1.47, 2.28
**Drug abuse**				
No	ref	ref	ref	ref
Yes	8.00***	7.19, 8.91	1.80***	1.43, 2.26
**Condom use**				
Never	ref	ref	ref	ref
Rarely	0.72***	0.63, 0.82	0.94	0.81, 1.09
Ever	0.58***	0.47, 0.70	0.92	0.74, 1.14
**Job title**				
Far from home	ref	ref	ref	ref
Risky environment	0.11***	0.05, 0.24	1.20	0.51, 2.81
Official	1.14**	0.60, 2.18	2.57**	1.28, 5.15
Unemployed	2.39***	1.25, 4.59	3.74**	1.86, 7.50
Student	0.25***	0.12, 0.52	1.47	0.67, 3.22
**Educational level**				
Illiterate	ref	ref	ref	ref
Elementary	1.13***	0.83, 1.53	1.07	0.75, 1.52
Junior	0.96*	0.71, 1.30	1.04	0.73, 1.47
High school	0.40***	0.29, 0.53	0.85	0.59, 1.21
Academic	0.12***	0.09, 0.16	0.48	0.34, 0.70
**Root of transmission**				
Injection drug	ref	ref	ref	ref
Unsafe sex (heterosexual)	0.06***	0.05, 0.07	0.21***	0.18, 0.25
Unsafe sex (homosexual)	0.16***	0.11, 0.23	0.64*	0.44, 0.93
Blood and blood products	1.11	0.66, 1.88	2.84**	1.57, 5.13
Vertical transfer	0.22***	0.16, 0.29	1.04	0.68, 1.58
High risk spouse	0.26***	0.20, 0.34	0.75	0.55, 1.02
HIV ^+^ Spouse	0.70***	0.59, 0.84	1.66**	1.29, 2.13
Job Exposure	0.004***	0.002, 0.01	0.023***	0.009, 0.06
Unknown	0.19***	0.17, 0.23	0.45***	0.38, 0.54

^*^p < 0.05, ^**^ *p* < 0.01, ^***^ *p* < 0.001

### Blinder-Oaxaca decomposition analysis

**Fig. 1:**
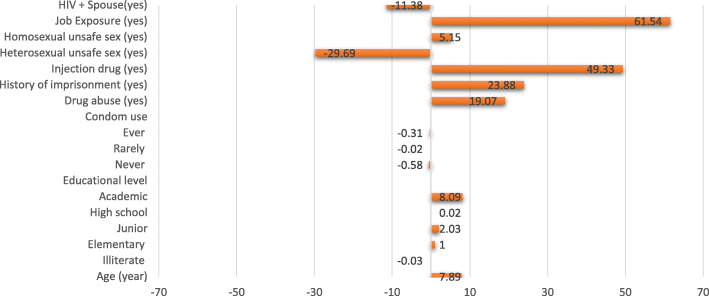
Contribution of determinants in total gap of differences in HIV infection among high-risk people, Tehran, Iran (2004–2018)

Tables 3 shows the results of the BO decomposition for determinants of HIV infection between female and male. The prevalence of HIV infection in female was 7.53% (95% CI: 6.90,8.20%), while the prevalence was 12.03% (95% CI: 11.51,12.54%) in male. The gap between female and male was − 4.50% (95% CI: − 5.33, − 3.70%). The results of the BO decomposition showed that − 136% of the gap between male and female could be explained by differences in the distribution of the variables included in the model. Differences in job exposure, drug abuse, history of imprisonment, injection drug, heterosexual unsafe sex, and having an HIV-positive spouse were the main determinants that affected the difference in the prevalence of HIV infection between the female and male. Figure 1 presents the contribution of determinants in the total gap of differences in HIV infection among high-risk people, Tehran, 2004 to 2018.

**Table 3:** Blinder-Oaxaca decomposition of the prevalence of HIV infection among high-risk people, Tehran, Iran (2004–2018)

	Prediction (%)	95% CI	Total gap (percent)^**a**^
Female	7.53***	6.90, 8.20	
Male	12.03***	11.51, 12.54	
**Total gap**	−4.50***	−5.33, −3.70	
***Due to endowment (explained)***			
**Age (year)**	−0.36***	−0.49, − 0.22	7.89
**Educational level**			
Illiterate	0.001	−0.008, 0.01	−0.03
Elementary	−0.05*	− 0.09, − 0.003	1.00
Junior	− 0.09*	− 0.16, − 0.02	2.03
High school	− 0.001	− 0.01, 0.01	0.02
Academic	− 0.36***	− 0.53, − 0.20	8.09
**Condom use**			
Never	0.03	−0.01, 0.07	−0.58
Rarely	0.001	−0.02, 0.02	−0.02
Ever	0.014	−0.02, 0.05	−0.31
**Drug abuse (yes)**	−0.86***	−1.25, − 0.46	19.07
**History of imprisonment (yes)**	−1.07***	− 1.50, − 0.70	23.88
Injection drug (yes)	−2.22***	−2.70, − 1.74	49.33
Heterosexual unsafe sex (yes)	1.34***	0.99, 1.68	−29.69
Homosexual unsafe sex (yes)	−0.23**	− 0.38, − 0.09	5.15
Job Exposure (yes)	− 2.77***	−3.35, − 2.19	61.54
HIV ^+^ Spouse (yes)	0.51	−0.03, 1.05	−11.38
*Sub-total of gap (explained part)*	−6.12***	−9.50, −2.73	136.00
***Due to response (unexplained)***			
**Age (year)**	−4.91	−14.82, 4.99	109.12
**Educational level**			
Illiterate	0.12	−0.14, 0.38	−2.66
Elementary	0.063	−0.35, 0.47	−1.40
Junior	−0.05	−0.75, 0.65	1.07
High school	−0.41	−1.56, 0.74	9.11
Academic	−1.91	−5.32, 1.49	42.44
**Condom use**			
Never	−0.31	−1.33, 0.72	6.78
Rarely	−0.12	−1.79, 1.54	2.74
Ever	0.23	−0.55, 1.00	−5.05
**Drug abuse (yes)**	−0.32	−2.54, 1.91	7.06
**History of imprisonment (yes)**	−4.06	−11.29, 3.17	90.22
Injection drug (yes)	1.72	−1.20, 4.63	−38.20
Heterosexual unsafe sex (yes)	−2.90	−7.63, 1.82	64.54
Homosexual unsafe sex (yes)	−0.28	−0.80, 0.25	6.15
Job Exposure (yes)	−0.56	−1.86, 0.73	12.54
HIV ^+^ Spouse (yes)	0.08	−0.07, 0.24	−1.82
*Sub-total of gap (Unexplained part)*	−13.62	−51.98, 24.75	302.65
***Interaction***	3.10**	0.94, 5.25	−68.86

^a^Calculated by dividing the determinant’s prediction value by the total gap (−4.5), ^*^ *p* < 0.05, ^**^ *p* < 0.01, ^***^ *p* < 0.001

The unexplained part of the difference between the female and male was 302.65% that may be attributed to differences in the coefficients of included determinants or other determinants that we were not included in the model. The share of the interaction component in the total gap between female and male was 68.86% (Table 3).

## Discussion

To develop effective HIV prevention and therapeutic interventions, it is important to identify the demographic characteristics of infected people. This study provides evidence about gender inequality in HIV infection and its responsible factors among high-risk people in Tehran. Our finding showed that there is a difference in the HIV infection between female and male. Male were more likely to get HIV infection than female. The difference in the manifestations of HIV infection among the female and male may have several individual and social reasons. This could be due to combined influences of inherent physiological factors (sex) and gendered social bias or through gendered social bias alone [22]. Where physiological sex differences interact with social factors, it defines different needs and influences exposure and vulnerability to health risks [22]. Specifically, in context of our study, this specific gender difference not only influence the acquisition of the virus but also the progression of the disease that policy efforts must address them to prevent risk outcome among at high-risk population. Also, some social factors such as social stigma and social network characteristics can increase likelihood of HIV risk behaviors [23–25] among male which can contribute to the observed gap between male and female. Living with HIV is along with social stigma in Iran, and based on the evidence this social stigma is higher among women than men [26, 27], because the community may think that those infected women especially those who are single may have premarital sex, deviant sexual behavior, and were infected via sexual relationships. Therefore, female are less likely to seek HIV testing services than men due to the profound social stigma associated with HIV, however, they usually more disclose their HIV status to their private network [28]. Another possible explanation is that Iranian male has a larger network size than female which can result in having more frequency of contact with people having HIV risk behaviors, receiving negative social support enhancing HIV risk behaviors and experiencing more risky behaviors which can make male more vulnerable to HIV infection than female [23–25, 29, 30]. Moreover, social norms related to male role in sexual relationships, such as being powerful, and determinator about condoms use behavior can led to unsafe sex and consequently HIV infection. Some qualitative studies among at risk female in Iran found that male have more control over condom use decisions than female as many of female participants believed that male have greater authority and domination in decision-making control over condom use and female are expected to yield to male desires for sex and condom use [11, 31]. This finding suggests that a gender-based HIV prevention intervention is necessary. Also, HIV-related interventions must consider gender stereotypes in Iranian society to reduce the social stigma and HIV prevalence gap between men and women. Our finding regarding the gender inequality in HIV infection is not consistent with previous studies [32–35]. A systematic review conducted in 2012, reported a significantly higher HIV prevalence among female compared to male who injects drugs [33]. One study was conducted in the USA, which enrolled 769 intravenous drug users, found that the gender of the participants was unrelated to their HIV status [32]. This controversy may be due to differences in methodological aspects, and social and cultural aspects among the studies.

We also found the main determinants of the observed difference in HIV infection between female and male. Our finding showed that job exposure, drug abuse, history of imprisonment, injecting drugs, heterosexual unsafe sex, and having an HIV-positive spouse were the main contributors to the difference in the prevalence of HIV infection between the female and male. Consistent with previous studies, our finding showed that male tended to have riskier sexual behavior, more use or injecting drugs, and history of imprisonment than female [7, 10, 14, 36]. These determinants may contribute to gender inequalities in HIV infection by providing a high-risk environment which increases the probability of HIV infection. One study showed that the majority of female involved in lower levels of jobs in health care, which increase risks of infection [37]. However, male generally works in environments with greater risk for job exposure [38]. In our study only 2.3% of people reported homosexual unsafe sex and nearly all (99.99%) of them were male. One explanation for the opposite effect of hetero and homo sexual unsafe sex on the gender difference in prevalence of HIV infection is that in a Muslim country, such as Iran, homosexuality is forbidden and banned by the religion and government, therefore this type of sexual behavior is very restricted and underreported. Therefore, any intervention in this term may result in identifying more HIV infected cases. As expected, the gap in HIV infection is reduced substantially when drug abuse, history of imprisonment, injecting drugs, heterosexual unsafe sex is taken into account. Our finding highlights that for HIV/AIDS prevention programs to be effective it is critical to be targeted and consider job characteristics, the history of drug abuse, history of imprisonment, and HIV risk behaviors of at-risk people especially men.

Our study had four limitations that should be considered when interpreting the study findings. First, we used the data that come from medical records of high-risk people that are volunteer to receive free counseling, testing, and treatment services; therefore, our results could be biased due to self-volunteer bias. Second, because our study design was cross-sectional, we are not able to establish any causal relationship between HIV infection and its main determinants included in the analysis. Third, the root of transmission was self-reported and may be reported with some degree of measurements error. Fourth, a proportion of people had missing data, we assume that the missing data are at random. However, to account for missing data, we used multiple imputations using chained equations.

## Conclusion

The findings can provide evidence for health policymakers and decision-makers to better planning and conducting gender-based preventive and screening programs to reduce the gender gap in HIV infection. Taking into consideration these gender differences is very important for effective HIV prevention and treatment interventions in a resource-limited setting, such as Iran. The results of the present study point to the importance of looking at multiple determinants when assessing gender inequality. Also, our finding highlights that policies and programs aimed at change the job exposure, drug abuse, history of imprisonment, injection drug, and heterosexual unsafe sex among the male may reduce the gap in HIV infection between female and male in Iran.

## Data Availability

The datasets used and/or analyzed during the current study are available from the corresponding author on reasonable request.

## Abbreviations

HIV: Human immunodeficiency virus
VCT: Voluntary Counseling and Testing
MI: Multiple Imputation
BO: Blinder-Oaxaca
CE: Coefficients
